# Supplementary material for: Induction of Resistance Against Sclerotinia sclerotiorum in Rapeseed by β-Ocimene Through Enhanced Production of Coniferyl Aldehyde
Source: Int J Mol Sci. 2025 Jun 13;26(12):5678. doi: 10.3390/ijms26125678 (PMC12193217; doi:10.3390/ijms26125678)
Supplement: Supplementary file 1 [file ijms-26-05678-s001.zip › ijms-3625774-supplementary.pdf]

The following 6 groups were included: untreated group (control), treated groups (totally 5 groups treated with 1 μM, 5 μM, 10 μM, 15 μM, and 20 μM β-ocimene, respectively). A 20 L desiccator was placed under direct sunlight for 2 hours following thoroughly wash. Prior to use, the desiccator was disinfected with 75% ethanol. With rapeseed placed in the desiccator, glass slides were heated to 45–50°C and coated with β-ocimene in a zigzag pattern to rapidly volatilize β-ocimene. The heated glass slides coated with β-ocimene at final concentrations of 1 μM, 5 μM, 10 μM, 15 μM, and 20 μM were then placed inside the container. The desiccator was sealed with a rubber band and transferred to an artificial climate chamber (light intensity of 100 mMol·m²·s⁻², relative humidity of 65%) for 12 hours before sample collection.

As shown in the figure, β-ocimene treatment induced the simultaneous gene expression of *BnaPDF1.2* and *BnaPR1*. Among the treatment groups, rapeseed treated with 15 μM β-ocimene exhibited the most significant co-expression of *BnaPDF1.2* and *BnaPR1*. Therefore, β-ocimene was treated at 15 μM in this study.

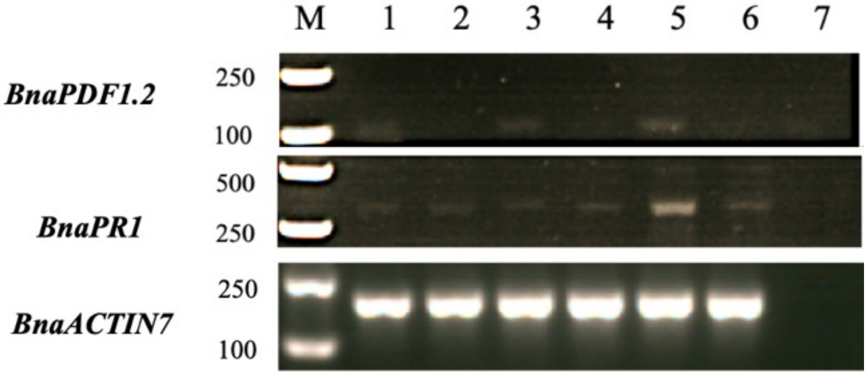

Figure S1. Determination of β-ocimene concentration treatment gradientn by RT-PCR

Note: M: DNAmarker, 1: control group, 2-6: PCR products of cDNA extracted from Brassica napus treated with five different concentrations of β-ocimene, 7: blank contro
